# The Use of Precision Feeding During the Lactation of a Traditional Breed, Iberian Pig, Improves Maternal Nutritional Status and Offspring Performance

**DOI:** 10.3390/biology15010033

**Published:** 2025-12-25

**Authors:** María Aparicio-Arnay, Antonio Gonzalez-Bulnes, Natalia Yeste-Vizcaino, Carlos Piñeiro, Beatriz Isabel

**Affiliations:** 1 Animal Data Analytics, S.L., C/Dámaso Alonso, 14, 40006 Segovia, Spain; carlos.pineiro@ada-animaldata.com; 2 Faculty of Veterinary Medicine, Universidad Complutense de Madrid, Ciudad Universitaria s/n, 28040 Madrid, Spain; bisabelr@ucm.es; 3 Faculty of Veterinary Sciences, Universidad Cardenal Herrera-CEU, CEU Universities, C/Tirant lo Blanc, 7, Alfara del Patriarca, 46115 Valencia, Spain; 4 Cuarte S.L., Grupo Jorge, Ctra. de Logroño km 9.2, Monzalbarba, 50120 Zaragoza, Spain; nataliayeste@cuartesa.com

**Keywords:** Iberian-breed, fatty pig, lactation, nutrition, precision-feeding, swine

## Abstract

The present study evaluated the impact of precision feeding using Electronic Sow Feeders (ESFs) during lactation on the nutritional status of Iberian sows and the performance of their offspring under intensive farming conditions. Fifty-three second-parity Iberian sows were assigned to three feeding systems: ESFs (PF), feeding ball (FB), and conventional feeders (CON). Results showed that PF sows had a significantly lower feed disappearance, without affecting body weight and condition, when compared to FB and CON groups (*p* < 0.05). Although milk yield did not differ significantly among groups, PF piglets exhibited higher weaning weights and average daily weight gains (ADWG), particularly during the second half of lactation (*p* < 0.05 and *p* < 0.001, respectively). Metabolic profiling revealed an improved glucose and lipid homeostasis in PF sows, with lower Non-Esterified Fatty Acids (NEFAs) levels and more favorable cholesterol profiles (*p* < 0.05 for all). No remarkable differences were observed in welfare biomarkers (salivary cortisol and alpha-amylase) among treatments. These findings suggest that precision feeding enhances feed efficiency, stabilizes maternal metabolism, and improves piglet growth in traditional swine breeds (“fatty pigs”) like the Iberian pig, offering a sustainable strategy for intensifying production without compromising animal welfare.

## Simple Summary

The use of electronic sow feeders (ESFs) during lactation in Iberian pigs results in increases in weaning weights of piglets concurrently with decreases in maternal feed expenses, which boosts the economic and environmental sustainability of production. The present trial also evidences improvements in metabolic traits of sows and piglets, of paramount importance in fatty breeds, without modifying welfare biomarkers.

## 1. Introduction

The Iberian pig is one of the main traditional swine breeds (also known as “fatty pigs”) worldwide. The Iberian breed, reared for centuries in extensive systems across the central and southwestern regions of the Iberian Peninsula (Spain and Portugal), is internationally recognized for the production of a unique highly priced dry-cured product, Iberian ham [1]. Currently, the increasing demand for this and other products derived from the breed (cured loin, spiced sausage, salami, bacon, and fresh pork) is driving a shift from traditional extensive management toward more intensive systems, based on direct adaptation of management strategies from lean commercial breeds, with the aim of increasing productive efficiency.

However, these strategies overlook the significant physiological and metabolic features of the Iberian breed, which has developed after centuries of exposure to extensive semi-feral conditions [1,2]. In brief, Iberian pigs exhibit slower growth rates and lower protein deposition than lean swine breeds [3], alongside a concomitant increase in voluntary food intake and a trend toward higher adiposity [4,5]. The ability to store excess fat in subcutaneous, visceral, and inter- and intramuscular depots during periods of food abundance allows pigs to cope with seasonal cycles of feasting and famine when reared in extensive systems [6,7]. The abundance and specific composition of inter- and intramuscular fat are, on the other hand, the basis for the superior quality of the Iberian pork products [1].

The Iberian pig is also characterized by lower reproductive efficiency than lean breeds, with lower prolificacy (a mean of 8.3 live piglets per farrowing) and longer weaning-to-conception intervals (around 8.4 days, which means around 2 farrowing per year) due to failures or delays in the resumption of estrus behavior and ovulatory activity after weaning [7]. Intensification of production has aimed to improve these traits in breeding sows, mainly by increasing reproductive management and implementing novel nutritional strategies. However, the metabolic features of traditional breeds imply more sensitivity to malnutrition than lean breeds, whether due to over- or underfeeding, or imbalances in diet composition [8].

Consequently, Iberian breeding sows are predisposed to develop insulin resistance and dyslipidemia in response to inadequate management during periods of nutritional challenge, such as lactation [9]. Malnutrition and inadequate metabolic states during lactation lead to disturbances in the growth of the litter [10] and the resumption of reproductive activity after weaning [11]. In fact, lactation is a challenging period for maternal homeostasis in all species because the lactating female must produce milk for the offspring while maintaining her own body condition and metabolism. Hence, maintaining an adequate nutritional status is crucial during lactation. In swine, this is even more critical in hyperprolific sows, since milk production for large litters requires substantial mobilization of body reserves, resulting in a catabolic state [12]. The scenario is the same in modern Iberian herds under intensive management, because the current trend of increasing prolificacy boosts milk demand during lactation and, thus, leads to a higher metabolic demand for the sow [13]. Lactation is also a challenging period for the piglet, as its health status, nutrition, and development during suckling determine its live weight at weaning, and its post-weaning and lifelong performance [14].

Adequate feeding and nutrition of prolific Iberian sows is therefore becoming increasingly necessary. The use of Electronic Sow Feeders (ESFs) is currently increasing in hyperprolific sows, as they allow the amount of feed offered to each sow to be adjusted depending on their individual needs [15,16]. This is of special interest during lactation, as this phase is characterized by a high metabolic demand and a high inter-individual variability of such demand [13]. Former studies of our team demonstrated that the use of ESF during the lactation of hyperprolific sows (Danbred breed; [17,18,19]) reduces feed disappearance from around 5 to 35 kg per sow and lactation, depending on adequacy of facilities and management, but also losses in body weight and condition, by around 4 kg, which suggests a better feed behavior. At the same time, the use of ESF improves litter weight at weaning from around 4 to 15 kg, depending again on type of facilities and management. Thus, the use of ESF decreases the cost of feed per weaned piglet in around EUR 0.70 under the current feed cost. Moreover, ESFs may be a key tool for improving feed efficiency, reducing nutrient wastage, and minimizing environmental impacts [15,16], which supports the sustainability of modern pig production by optimizing resource use without compromising productivity or animal welfare.

However, to the best of our knowledge, no previous studies have investigated the use of ESFs in Iberian sows or other traditional breeds. Traditional breeds, with a background of extensive semi-feral rearing, have different feeding patterns from modern strains, with exploratory behavior and the consumption of more frequent but smaller meals throughout the day. We hypothesized that offering multiple small feed rations throughout the day, a pattern adequately supported by the use of ESFs, could improve the nutritional and metabolic status and thus the productive yields of Iberian sows and their offspring. Hence, the present study aimed to assess the effects of using ESFs on the productive performance of lactating Iberian sows and their offspring, reared under intensive farm conditions.

## 2. Materials and Methods

### 2.1. Ethics Statement

The research was conducted on a commercial farm (La Frondosa farm, Garrapinillos, Zaragoza, Spain) according to the European Union Directive and the Spanish Policy for Animal Protection RD53/2013. The Committee of Ethics in Animal Research of the Universidad Complutense de Madrid (UCM) approved the experimental procedures (CEEAH2788M2).

### 2.2. Animals, Experimental Treatments and Management

The study involved 53 Iberian sows (second parity). All the sows were individually allocated to the same type of farrowing stalls from the week before parturition to the day of weaning, but were distributed in three groups based on the use of different types of feeders. The first group (Group PF; n = 17 sows) was fed with electronic sow feeders (Gestal SOLO+, Jyga Technologies Inc., Saint-Lambert-de-Lauzon, QC, Canada; six rations per day at 5 AM, 8 AM, 11 AM, 2 PM, 5 PM, and 8 PM); the second group (Group FB, n = 18) was fed with feeding-ball equipment (Feeding Ball, Rotecna, Agramunt, Spain; feed allowed the whole day); the third group (Group CON; n = 18) was fed with traditional feeders (SB, Rotecna, Agramunt, Spain; feed given at 8 AM and 2 PM). The amount of feed provided could be manually adjusted in the CON group by farm staff based on the sow’s feed intake from the previous day. In contrast, feed adjustments were made automatically by the device in the FB and PF groups, allowing sows the opportunity to consume up to 20% more feed at each feeding event.

The management of sows and their offspring followed standard intensive farm practices, with the animals being housed indoors under controlled temperatures. Productive data were recorded per sow at birth, including the total number of live and stillborn piglets and litter weight. Feed behavior, feed disappearance, and changes in body weight and condition during lactation were studied in all the sows (n = 53), but a more comprehensive study was performed in approximately half of them (n = 29; nine sows from group PF, 10 sows from group FB, and 10 sows from group CON), including productive performance, milk traits (quantity and quality), and postnatal growth of the piglets. The study also evaluated the metabolic and welfare status of sows and piglets during lactation.

### 2.3. Feed Disappearance and Changes in Body Weight and Condition of Sows

Feed disappearance was individually and daily recorded for all the sows involved in the trial (n = 53; manually in the groups FB and CON and automatically in the group PF) from the day of farrowing to the day of weaning.

The weight and body condition (back-fat depth) of all sows were measured at the beginning (i.e., one week before parturition) and at the end of the study (i.e., the day of weaning, approximately four weeks after delivery). Back-fat depth was measured at the P2 point, which lies on the right side of the animal at 4 cm from the midline and transversal to the head of the last rib [20], using an ultrasound equipment fitted to a multi-frequency linear array probe (ProVetScan SF2 Wireless scanner, NewVetec, Leon, Spain). The initial and final body weights and backfat depths of all sows were used to calculate the losses in body weight and condition during lactation. Absolute body weight loss (ABWL) was calculated as the difference between weight after farrowing (initial weight before farrowing minus litter weight at birth) and weight at weaning, while relative body weight loss (RBWL) was obtained by dividing ABWL by the weight after farrowing, as previously described in other studies [21,22].

### 2.4. Quantity and Quality of Milk

Milk production was estimated in a subgroup of 29 sows using the weigh–suckle–weigh (WSW) technique [23] at 14 and 28 days of lactation. In brief, piglets were separated from the sow for a two-hour period to prevent suckling and were individually weighed just before being returned to the sow. After that, the piglets were allowed to suckle freely and were weighed again immediately after they were satiated. The difference in body weight before and after suckling was used to estimate milk production during the two-hour period.

Milk composition was assessed at 14 days of lactation in samples (20 mL) obtained by individual milking of the sows. In brief, the milk was collected from all functional glands by hand-milking after the sows were injected with 20 I.U. oxytocin (Oxitocina Diana, Super’s Diana S.L, Barcelona, Spain). Immediately after milking, samples were stored at −80 °C until assayed for lactose, protein, fat content, and dry matter using an IR spectrophotometer (MilkoScan ™ 7RM, Foss Iberia, Barcelona, Spain).

### 2.5. Postnatal Growth of the Piglets

All living piglets born in the subgroup of 29 sows were used for individual assessment of postnatal growth. After individual identification with electronic ear tags, within-group cross-fostering and allocation to mothers at a rate of approximately 9 piglets/sow, a total of 265 piglets were selected for the trial. Creep feeding was offered to all the piglets from 14 days of age.

These piglets were individually weighed at birth and at weaning, at 28 days old. The average daily weight gain (ADWG) for the interval of 0–28 days was individually determined using the formula ([final weight—initial weight]/number of days). At 14 days after farrowing, a subset of 116 piglets was selected to be weighed among a representative population (n = 4 in each litter, avoiding the smallest and largest piglets; all piglets were weighed). The ADWG for the intervals 0–14 and 14–28 was determined in these 116 piglets using the formula previously explained.

### 2.6. Metabolic and Welfare Status of Sows and Piglets

The metabolic and welfare status of all sows was evaluated at the beginning of the study, 14 days after farrowing, and at weaning (28 days after farrowing). Additionally, a subset of 116 piglets was assessed for metabolic and welfare parameters at 14 days postpartum and again at weaning. The metabolic status of both sows and piglets was determined using blood samples drawn by puncturing the vena cava cranealis using EDTA vacuum tubes (Vacutainer Systems Europe, Meylan-Cedex, France). Immediately after collection, the blood samples were centrifuged at 1500 *g* for 15 min, and the plasma was separated and stored at −80 °C until analysis of metabolic biomarkers. Plasma concentrations of glucose (glucose and fructosamine), protein (total protein content), and lipid metabolism (total cholesterol, high-density lipoprotein cholesterol [HDL-c], low-density lipoprotein cholesterol [LDL-c], triglycerides and Non-Esterified Fatty Acids (NEFAs)) were assessed using a clinical analyzer (Konelab 20i Chemistry Analyzer, Thermo Fisher Scientific, Madrid, Spain).

Welfare status was determined by assessing salivary stress biomarkers (cortisol and alpha-amylase). Saliva samples were collected from the sows and piglets at weaning by offering a polypropylene sponge (Koronis ref. SKU 030004, La Griega E. Koronis, Madrid, Spain) clipped to a flexible thin metal rod to be chewed by the pigs. Immediately, the sponges were placed into Salivette^®^ tubes (SARSTEDT S.A.U., La Roca del Vallès, Spain), centrifuged at 3500 *g* for 10 min, and stored at −80 °C until analyzed. Subsequently, commercial assays were used to quantify salivary cortisol (Expanded Range High Sensitivity Salivary Cortisol Enzyme Immunoassay Kit, Salimetrics, Carlsbad, CA, USA) and alpha-amylase content (Salivary alpha-amylase kinetic enzyme assay Kit, Salimetrics, Carlsbad, CA, USA) [24]. Saliva samples from piglets at 14 days of age were collected with sterile dry swabs supplied in shockproof round-bottom polypropylene tubes, with a label sealing the cap, Ø13 × 165 mm, and sterilized with ethylene oxide, ref. 300261 (Deltalab, Plz. Verneda 1, Pol Ind La Llana, 08191 Rubí, Barcelona, Spain) and stored at room temperature for further analysis of cortisol concentration using the technique described by Chacón et al. [25] and based on an enzyme immunoassay technique (ELISA; Expanded Range High Sensitivity Salivary Cortisol Enzyme Immunoassay Kit, Salimetrics, Carlsbad, CA, USA).

### 2.7. Statistical Analysis

Data were analyzed using SPSS 22.0 (IBM, New York, NY, USA). The sow or piglet was considered the experimental unit, depending on the litter or individual piglet assessment. One-way ANOVA and Duncan’s post hoc test were used to assess the possible effects of the feeding system (CON vs. PF vs. FB) on feed disappearance, weight, and metabolic, welfare, and productive conditions of sows. Changes over time in the developmental traits (changes in weight over time), and the metabolic and welfare status of the piglets were assessed by ANOVA for repeated measures (split-plot ANOVA), after using a Kolmogorov–Smirnov test for verification of normal distribution. All results are expressed as mean ± standard deviation (SD), with *p* < 0.05 representing statistical significance and 0.1 > *p* > 0.05 denoting a trend.

## 3. Results

### 3.1. Productive Data at Farrowing

There were no significant differences in the mean number of total, born alive, or stillborn piglets among sows in Groups PF, FB, and CON. Concomitantly, there were no differences in the individual piglet weight or total litter weight at farrowing (Table 1).

### 3.2. Feed Disappearance and Changes in Body Weight and Condition of Sows

There were no differences among the groups in body weight and backfat depth of the sows at the beginning of the trial (Table 2). Assessment of body weight at weaning showed that sows in the FB group had significantly higher weight losses compared to the other groups (approximately 10 kg), whereas sows in the Group CON gained approximately 6 kg and sows in the Group PF remained stable. However, the assessment of absolute and relative body weight losses during lactation (ABWL and RBWL, respectively) showed no significant differences among the groups. Concomitantly, there were no significant differences among groups in the backfat depth of the sows at weaning.

Despite the lack of differences in the evolution of body weight and backfat, the total feed disappearance during lactation was significantly lower in Group PF (104.72 ± 1.17 kg) than in Groups FB and CON (110.79 ± 1.14 and 109.03 ± 1.14 kg, respectively; *p* < 0.05).

### 3.3. Quantity and Quality of Milk

There were no significant differences in the amount of milk produced at either mid- (Day 14) or late lactation (Day 28), as shown in Table 3, despite a trend of increasing milk production over time in Groups PF and CON, while it decreased in Group FB. It is important to highlight the high individual variability in milk production in all the sows, especially in Group FB at the end of lactation.

Assessment of the milk composition at 14 days of lactation (Table 4) showed that sows in Group FB had a significantly higher amount of protein (*p* < 0.01) and a numerically higher amount of dry matter (*p* = 0.07) than sows in Group CON.

### 3.4. Postnatal Growth of the Piglets

No significant differences were observed among the experimental groups in the number of piglets weaned on Day 28 (Table 5). However, the assessment of individual piglet weaning weights showed that the heaviest piglets were in the PF group compared to the FB and CON groups (*p* < 0.001 and *p* < 0.05, respectively). Such differences were related to significantly higher individual weight increases and, thus, a higher Average Daily Weight Gain (ADWG) in PF piglets than in FB and CON piglets (*p* < 0.001 and *p* = 0.001, respectively). Consequently, the increase in litter weight over lactation (0–28 days) was significantly different between groups PF and FB (*p* < 0.05), but only a trend was found when comparing groups PF and CON (*p* = 0.054). Thus, there was also a significant difference in the Average Daily Gain per litter (kg/day) between the PF group and both the FB and CON groups (*p* < 0.05). Therefore, at weaning, the litter weight was approximately 10 kg heavier in the PF group than in the FB and CON groups. However, the statistical analysis did not show that these differences were statistically significant.

The assessment performed in the subset of 116 piglets confirmed a higher ADWG in the PF group than in the FB and CON groups (Table 6). However, assessment of the first and second parts of pregnancy (0–14 days and 14–28 days, respectively) showed different growth patterns. There were no significant differences among groups in the individual ADWG for the first part of lactation and thus in the individual weight at mid-lactation (Day 14; Group PF: 4.22 ± 0.13; FB: 4.04 ± 0.13; CON: 4.06 ± 0.13). There was a trend for differences in the ADWG among groups in the 14–28 days interval (*p* = 0.09), with significant differences between PF and FB (*p* = 0.016).

### 3.5. Metabolic and Welfare Status of Sows and Piglets

The assessment of plasma concentrations of biomarkers for glucose, lipid, and protein metabolism in the sows at the start of the trial (Day 0), mid-lactation (Day 14), and weaning (Day 28) showed significant differences among groups in some of these biomarkers (Table 7). On Day 14, significant differences were observed in the plasma LDL-C concentrations among the group CON (53.43 ± 9.00), PF (43.46 ± 6.09; *p* < 0.05), and FB (40.36 ± 8.89; *p* < 0.005). On Day 28, differences were found in glucose and lipid metabolism among the groups, with the PF group showing the highest glucose levels and the CON group having the highest values for cholesterol, HDL-C, LDL-C, and triglycerides. Finally, Non-Esterified Fatty Acids (NEFAs) were found to be significantly higher in the FB group than in the PF group.

In piglets, significant differences were observed only on Day 14 (Table 8). Glucose levels were significantly higher in the FB group than in the CON group, with the PF group showing intermediate values. In contrast, lipid biomarkers (triglycerides and HDL-C) were significantly higher in the CON group than in the PF group (*p* < 0.05), with the FB group having intermediate values.

The assessment of salivary welfare biomarkers in sows during lactation showed no differences among the groups at any time point (farrowing, 14 days of lactation and weaning; Table 9).

In piglets, the salivary stress biomarkers (Table 10) indicate a significantly higher level of cortisol in piglets born from PF sows at Day 14 when compared to the other groups (*p* < 0.05). Additionally, amylase concentrations at Day 28 were significantly higher in piglets from the FB group compared to those in the CON and PF groups (*p* < 0.001).

### 3.6. Economic Impact

The analysis of feed disappearance per kg of weaned piglet (Table 11) also demonstrated better results for the PF group compared with the FB (*p* = 0.045), and showed no differences between PF and CON. However, no statistically significant differences were found between groups in the analysis of total feed intake per weaned piglet—only numerical differences in favor of the PF group.

## 4. Discussion

The results of the present study indicate that the implementation of precision feeding in lactating Iberian sows may be associated with a reduction in maternal feed expenses without compromising the body condition or welfare status of the sow, and may even improve the metabolic and productive traits of both the female and her piglets.

The present study compared three groups of lactating sows fed with three different types of feeders; Group PF was fed with precision electronic feeders, Group FB was fed with feeding-ball equipment, and Group CON was fed with traditional feeders. The sows were able to adjust their feed intake in the first two feeders, but precision feeding also distributes the meal in several portions over the day. Our results indicate that total feed use during lactation was approximately 4–6 kg lower in the group fed with electronic feeders than in the other groups. However, the assessment of possible changes in body weight and backfat depth during lactation showed no significant differences among sows in these three groups in either the absolute or relative body-weight loss (ABWL and RBWL, respectively) or in the trajectory of backfat depth. Hence, the lower feed use when using precision feeding may be mainly due to a decrease in feed wastage, rather than a decrease in feed intake.

Such a finding is especially interesting given the intrinsic characteristics of the Iberian pig and other fatty breeds. Fatty pigs have a higher voluntary food intake and a higher trend toward adiposity than lean swine breeds due to a gene polymorphism for the leptin receptor (*LEPR*), which is similar to the syndrome of leptin resistance described in human medicine [4,26,27,28]. Therefore, fatty pigs do not experience satiety are used to eating all the available feed; concomitantly, these pigs display strong exploratory feeding behavior. We can hypothesize that the pattern of several meals throughout the day provided by precision feeding, which delivers more frequent and smaller portions than the other feeders, may better align with the exploratory feeding behavior typical of the Iberian breed and may explain the reduction in feed wastage observed in our trial. In this regard, some authors have reported that sows fed ad libitum with precision feeders during lactation spend more time interacting with the trigger mechanism of the feeder, which reflects increased exploratory activity and which was associated with an improved welfare status [16]. Moreover, eating more times per day in small quantities, besides decreasing feed wastage, improves meal digestibility [29].

In any case, an initial finding to highlight in our study is that the differences in feed intake found in the sows fed with precision feeders, which did not affect the body weight and condition of the sows, have a direct economic impact on the farm’s output. In our study, the PF group saved 6.07 kg and 4.31 kg per sow and lactation when compared with groups FB and CON, respectively. Such an amount, at a current feed cost of 0.337 EUR/kg, represents cost savings of EUR 2.05 and EUR 1.45 per sow and lactation, respectively, and, in turn, total savings of EUR 4920 and EUR 3485 per year in a farm of 1000 Iberian sows (plus greater environmental sustainability by decreasing feed wastage). Thus, the findings of our seminal study addressing the impact of precision feeding in lactating Iberian sows are consistent with those previously reported in hyperprolific lactating sows [15,16,17,18,19].

Our results also indicate that, alongside the lack of differences in body weight and backfat depth among groups, the lower feed expenditure in the precision feeding system did not affect the welfare status of the sows (as measured by salivary cortisol and alpha-amylase). Conversely, it should be noted that the use of precision feeding systems improved the metabolic status of the sows during lactation.

In this regard, the assessment of the metabolic status of the sows showed significant differences among groups, which were not found in previous studies with commercial lean breeds [18]. At the beginning of the study, there were similar values among groups for all the plasma metabolic biomarkers. Subsequently, however, sows in the CON group, fed with traditional feeders, showed significantly higher plasma concentrations of LDL-C at mid-lactation than sows in the PF and FB groups; at weaning, these differences were also found in total cholesterol and triglycerides. Elevated plasma concentrations of triglycerides and cholesterol—a dyslipidemic profile—in non-obese individuals, such as the sows in our study, are indicative of mobilization of fat reserves [11]. Hence, higher plasma concentrations of cholesterol and triglycerides in the CON group may indicate an inadequate nutritional state throughout lactation. In addition, significantly higher concentrations of non-esterified fatty acids (NEFA) were found in the FB group than in the PF group at weaning; NEFA levels are also a marker of lipid catabolism, and elevated circulating NEFA concentrations are indicative of negative energy balance with deficient glucose metabolism and lipomobilization. As a result, the differences in NEFA concentrations between the PF and FB groups suggest that several controlled meals throughout the day favour and optimize the energy metabolism of the Iberian sows.

Moreover, in spite of the decrease over lactation, the PF group showed significantly higher plasma glucose levels at weaning than the other two groups. Values in the PF group were about 13% lower than those recorded at the beginning of the study, whilst values in the FB and CON groups decreased by around 48% and 30.8%, respectively. In the absence of differences in fructosamine concentrations (an index indicative of preceding glucose levels, over the previous 2–3 weeks [30], and therefore of long-term over- or under-nutrition), these differences suggest improved short-term glucose availability in the PF group. This may be potentially due to the more frequent feed allowance provided to the sows fed with precision feeding systems and may be especially important in fatty sows, considering the trend for insulin resistance and impaired glucose tolerance in these breeds [31].

In summary, these results support that the use of precision feeding, providing several meals throughout the day, better aligns with the metabolic characteristics of the fatty breeds and improves the homeostatic state of lactating sows.

The assessment of the developmental patterns and metabolic and welfare status of the piglets also showed positive effects from the implementation of precision feeding. Our results indicate that, from similar litter size and birth weight at farrowing and similar litter size at weaning, the piglets in the PF group achieved significantly higher weaning weights than piglets in groups FB and CON (around 0.860 kg and 0.630 kg higher, respectively) due to a higher ADWG during lactation. This outcome also has a direct economic impact on the farm’s output, reinforcing the cost savings in maternal feed, since the cost of producing a weaned piglet was EUR 0.99 and EUR 1.22 higher in the FB and CON groups than in the PF group, respectively. Such difference, in a farm with 1000 sows and weaning around 19,200 piglets annually, would result in cost savings of approximately EUR 19,000 and 23,400 when comparing the PF group with the FB and CON groups, respectively. In such a farm of 1000 sows, we would need to set up around 300 ESF at the maternity facilities. It is more difficult to calculate the initial capital investment required for installing ESF because depends on the brand and model of the ESF and the characteristics of the farm. However, at a current cost of around EUR 500 per device, the initial investment would be around EUR 150,000. Such cost would be compensated in approximately 6 years considering only cost savings in maternal feeding and extra benefits of higher weaning weights. Again, these findings of our study are consistent with previous data reported in hyperprolific sows [15,16,17,18,19]. Also, it is important to remark other advantages as labor time saving and the indirect benefits of the production of heavier piglets at weaning, because it is associated with improved growth performance in subsequent phases, reduced nutritional complexity and feed costs, and lower mortality rates during nursery and growing periods. Additionally, larger piglets reach slaughter weight sooner and exhibit superior carcass quality, reinforcing the long-term benefits of this feeding approach [14,19,32,33,34].

The primary determinants of the growth rate of piglets during lactation are the maternal milk production, which is modulated by the intensity and efficiency of the suckling behavior of the piglet and the nutrient composition of the milk [35,36,37,38]. Concomitantly, providing piglets with creep feed from 14 days of lactation, as in our study, increases piglet body weight at weaning [39].

Some authors reported that milk production increases when sows spend more time standing [36], which would be consistent with ad libitum feeding systems like the one used in the PF group, where more frequent meals stimulate the sows to stand up more frequently. However, other authors failed to find differences in the amount of time that sows fed using traditional feeders or ad libitum systems spent standing [15]. In our study, despite numerical differences, there were no significant differences in milk production among feeding systems; however, it should be noted that only a single-point measurement was performed and that the data were affected by high individual variability, especially around weaning.

There is a positive correlation between milk production and lactose [38], and some authors reported that lactose content increases with feeding management whereby the sow eats more times per day at a slow speed [29]. These findings are supported by our previous results in commercial lean lines in which sows fed with precision feeding systems showed increased lactose content [17]. However, in the present study, these differences were not found. Only significantly higher protein values were observed in the FB group; previous studies have shown that protein concentration negatively correlates with milk production and growth of both piglets [40] and babies [41].

An assessment of stress biomarkers showed a higher cortisol level in PF piglets at Day 14 of lactation, which was no longer observed at weaning, and a higher alpha-amylase level in FB piglets at weaning. Such findings around weaning, besides numerically lower milk production supported by significantly higher protein content, may suggest a possible relationship with the lower ADWG of piglets in this group due to the negative effects that stress has on growth performance [42]. An assessment of the homeostatic status of the piglets showed that the CON group had lower blood glucose levels than in the FB group, with the PF group showing intermediate values, and higher levels of lipid biomarkers (triglycerides and HDL-C) than in the PF group, with group FB having intermediate values. Keeping in mind that metabolic parameters of suckling piglets rely on maternal milk composition, such data may support differences in milk production and composition among groups.

## 5. Conclusions

The use of precision feeding in lactating fatty sows decreases maternal feed expenses and increases weaning weight of the piglets, boosting economic and environmental sustainability, similarly to earlier reports in lean commercial lines. However, further studies are needed to determine possible improvements in the reproductive performance of sows and the productive efficiency of piglets after weaning. The present trial also demonstrates improvements in the metabolic traits of both sows and piglets without affecting their welfare traits, which is of paramount importance in fatty breeds and paves the way for future studies to dissect metabolic changes.

## Figures and Tables

**Table 1:** Mean (±SD) number of piglets born alive and stillborn, and weight of individual piglets and litters at birth, in sows fed with Electronic Sow Feeders (PF), feeding ball (FB), or traditional feeders (CON) during the lactation period.

Group	Born Alive (n)	Stillborn (n)	Individual Piglet Weight (kg)	Litter Weight (kg)
**PF**	10.11 ± 0.54	1.00 ± 0.00	1.43 ± 0.24	13.47 ± 1.32
**FB**	9.20 ± 0.51	1.00 ± 0.00	1.49 ± 0.32	13.86 ± 2.22
**CON**	9.30 ± 0.51	1.00 ± 0.00	1.51 ± 0.25	14.16 ± 1.65

**Table 2:** Mean values (kg ± SD) and *p*-values of initial and final body weight and backfat depth (IBW and FBW, kg, and IBFD and FBFD, mm) and absolute and relative body weight losses during lactation (ABWL and RBWL) of sows fed with Electronic Sow Feeders (PF), feeding ball (FB) and traditional feeders (CON).

Group	IBW	IBFD	FBW	FBFD	ABWL	RBWL
**PF**	158.92 ± 20.60	36.35 ± 10.12	157.41 ± 19.80 ^a^	31.02 ± 7.45	−2.10 ± 4.85	−0.01 ± 0.03
**FB**	154.06 ± 11.43	37.36 ± 8.85	144.67 ± 16.97 ^b^	31.91 ± 4.32	−9.39 ± 9.96	−0.06 ± 0.07
**CON**	150.00 ± 41.75	35.76 ± 8.31	156.39 ± 13.41 ^a^	31.92 ± 5.22	−6.38 ± 3.78	−0.84 ± 3.49

Different superscripts denote statistical differences (a ≠ b: *p* < 0.05).

**Table 3:** Mean values (g ± SD) and range (within parentheses) for milk production (mL) in sows fed with electronic sow feeders (PF), feeding ball (FB) and traditional feeders (CON) during lactation.

Group	Day 14	Day 28
**PF**	778.89 ± 363.02 (370–1490)	1218.00 ± 917.26 (80–2780)
**FB**	824.00 ± 557.70 (280–2240)	1031.00 ± 862.60 (0–3080)
**CON**	754.00 ± 260.78 (330–1110)	1141.00 ± 734.11 (0–2310)

**Table 4:** Mean values (% ± SEM) and *p*-values for milk components at 14 days in sows fed with electronic sow feeders (PF), feeding ball (FB), and traditional feeders (CON) during lactation.

Group	Dry Matter	Protein	Fat	Lactose
**PF**	18.85 ± 1.33	4.93 ± 0.14	5.96 ± 1.11	5.83 ± 0.12
**FB**	20.69 ± 3.16	5.23 ± 0.72 ^c^	7.40 ± 2.29	5.61 ± 0.32
**CON**	18.31 ± 1.60	4.54 ± 0.27 ^d^	5.90 ± 1.39	5.83 ± 0.16

Different superscripts denote statistical differences (c ≠ d: *p* < 0.01).

**Table 5:** Mean values (±SD) of total number of weaned piglets, total litter weight at weaning and individual weaning weight (kg), individual weight increase (kg), and individual Average Weight Daily Gain (kg/day), of piglets from sows fed with electronic sow feeders (PF), feeding ball (FB) and traditional feeders (CON) during lactation.

Group	Piglets Weaned	Individual Piglet Weight at Weaning	Individual Weight Increase (kg)	Individual ADWG (kg/Day)	Litter Weight (kg)
**PF**	8.67 ± 0.50	7.41 ± 1.21 ^ag^	5.98 ± 1.26 ^eg^	0.214 ± 0.05 ^g^	64.21 ± 7.05
**FB**	8.30 ± 1.06	6.55 ± 1.51 ^h^	5.06 ± 1.36 ^h^	0.159 ± 0.07 ^h^	54.40 ± 10.87
**CON**	8.10 ± 0.88	6.78 ± 1.33 ^b^	5.26 ± 1.10 ^f^	0.172 ± 0.07 ^h^	54.92 ± 9.21

Different superscripts denote statistical differences (a ≠ b: *p* < 0.05; e ≠ f: *p* < 0.005; g ≠ h: *p* < 0.001).

**Table 6:** Mean values (±SD) of litter weight increase (kg) and Average Weight Daily Gain of piglets (kg/day) from 0 to 14 days, from 14 to 28 days, and from 0 to 28 days of piglets from sows fed with electronic sow feeders (PF), feeding ball (FB), and traditional feeders during lactation (CON).

Group	Litter Weight Increase (kg) 0–14 Days	ADWG (kg/day) 0–14 Days	Litter Weight Increase (kg) 14–28 Days	ADWG (kg/day) 14–28 Days	Litter Weight Increase (kg) 0–28 Days	ADWG (kg/day) 0–28 Days
**PF**	2.75 ± 0.46	0.197 ± 0.03	3.17 ± 0.84	0.240 ± 0.06 ^a^	50.73 ± 6.97 ^a^	1.937 ± 0.27 ^a^
**FB**	2.57 ± 0.60	0.184 ± 0.04	2.74 ± 0.82	0.197 ± 0.06 ^b^	40.54 ± 9.17 ^b^	1.499 ± 0.32 ^b^
**CON**	2.66 ± 0.80	0.190 ± 0.06	2.89 ± 0.85	0.219 ± 0.06	40.75 ± 9.27	1.555 ± 0.34 ^b^

Different superscripts denote statistical differences (a ≠ b: *p* < 0.05).

**Table 7:** Mean values (±SEM) and *p*-values of plasma concentrations for parameters of glucose, lipids, and protein metabolism in sows fed with electronic sow feeders (PF), feeding ball (FB), and traditional feeders (CON) during lactation.

	Day	Glucose (mg/dL)	Fructosamine (µmol/L)	Cholesterol (mg/dL)	HDL-C(mg/dL)	LDL_C (mg/dL)	Triglycerides (mg/dL)	Proteins (g/dL)	NEFA-HR (mg/dL)
**PF**	0	112.89 ± 12.74	283.23 ± 21.94	74.00 ± 19.26	25.29 ± 7.29	44.04 ± 9.48	22.52 ± 11.76	7.65 ± 0.22	0.057 ± 0.03
	14	109.03 ± 15.78	285.30 ±17.54	87.62 ± 7.30	31.11 ± 4.54	43.46 ± 6.09 ^a^	17.79 ± 6.04	7.71 ± 0.58	0.163 ± 0.23
	28	98.48 ± 14.47 ^cg^	264.11 ± 17.71	133.51 ± 18.66 ^e^	56.82 ± 4.36 ^a^	55.07 ± 10.42 ^a^	23.98 ± 7.54 ^a^	7.51 ± 0.40	0.295 ± 0.36 ^a^
**FB**	0	138.55 ± 36.69	277.96 ± 20.62	76.86 ± 13.77	24.71 ± 3.40	45.15 ± 12.05	24.03 ± 12.21	7.63 ± 0.37	0.149 ± 0.16
	14	117.44 ± 31.54	283.55 ± 20.39	81.51 ± 18.33 ^a^	30.18 ± 10.21	40.36 ± 8.89 ^e^	19.84 ± 6.90	7.35 ± 0.35	0.189 ± 0.18
	28	72.49 ± 9.10 ^h^	252.53 ± 27.00	126.06 ± 17.26 ^g^	47.95 ± 10.60 ^g^	56.31 ± 6.76	38.79 ± 13.80 ^b^	7.09 ± 0.50	0.769 ± 0.34 ^b^
**CON**	0	114.72 ± 16.06	267.73 ± 20.58	88.75 ± 18.32	24.77 ± 9.29	56.66 ± 14.04	24.02 ± 11.95	7.67 ± 0.46	0.086 ± 0.05
	14	121.15 ± 48.95	282.91 ± 26.47	103.29 ± 19.47 ^b^	35.08 ± 6.04	53.43 ± 9.00 ^fb^	26.65 ± 15.41	7.65 ± 0.37	0.189 ± 0.13
	28	79.34 ± 13.78 ^d^	263.57 ± 14.61	168.28 ± 23.01 ^hf^	66.57 ± 6.91 ^bh^	66.41 ± 10.32 ^b^	37.36 ± 12.14	7.34 ± 0.52	0.636 ± 0.36

Different superscripts at same time points denote statistical differences (a ≠ b: *p* < 0.05; c ≠ d: *p* < 0.01; e ≠ f: *p* < 0.005; g ≠ h: *p* < 0.001).

**Table 8:** Mean values (±SEM) and *p*-values of plasma concentrations for parameters of glucose, lipids and protein metabolism in piglets from sows fed with electronic sow feeders (PF), feeding ball (FB), and traditional feeders (CON).

	Day	Glucose (mg/dL)	Fructosamine (µmol/L)	Cholesterol (mg/dL)	HDL-C (mg/dL)	LDL_C (mg/dL)	Triglycerides (mg/dL)	Proteins (g/dL)	NEFA-HR
**PF**	14	149.48 ± 12.92	180.32 ± 15.23	137.49 ± 26.25	48.24 ± 6.23 ^a^	63.88 ± 17.65	64.93 ± 20.43 ^a^	5.55 ± 0.43	0.218 ± 0.08
	28	134.50 ± 13.00	202.75 ± 18.48	136.01 ± 28.36	50.08 ± 7.02	65.18 ± 17.63	56.43 ± 23.84	5.22 ± 0.37	0.323 ± 0.144
**FB**	14	154.79 ± 17.28 ^a^	187.28 ± 17.53	138.02 ± 26.81	49.63 ± 5.17	60.09 ± 15.27	79.76 ± 37.58	5.57 ± 0.73	0.265 ± 0.12
	28	134.76 ± 16.37	205.82 ± 22.92	137.16 ± 34.08	49.93 ± 11.92	61.53 ± 13.60	69.35 ± 38.80	5.23 ± 0.65	0.324 ± 0.14
**CON**	14	144.38 ± 20.52 ^b^	186.62 ± 22.19	150.36 ± 38.04	51.95 ± 7.48 ^b^	68.42 ± 20.48	86.30 ± 42.68 ^b^	5.37 ± 0.40	0.260 ± 0.11
	28	130.30 ± 44.78	195.30 ± 51.07	150.90 ± 37.64	51.52 ± 15.39	66.31 ± 25.53	65.17 ± 39.18	5.10 ± 1.27	0.341 ± 0.17

Different superscripts at same time points denote statistical differences (a ≠ b: *p* < 0.05).

**Table 9:** Cortisol and alpha-amylase concentration at farrowing, 14 days after farrowing, and at weaning, in sows fed with electronic sow feeders (PF), feeding ball (FB) and traditional feeders (CON).

Group	At Farrowing		14 Days After Farrowing		At Weaning	
	Cortisol (μg/dL)	Alpha-Amylase (U/L)	Cortisol (μg/dL)	Alpha-Amylase (U/L)	Cortisol (μg/dL)	Alpha-Amylase (U/L)
**PF**	0.12 ± 0.07	2635.87 ± 3931.29	0.18 ± 0.10	309.52 ± 300.52	0.09 ± 0.05	1974.32 ± 2934.64
**FB**	0.09 ± 0.05	3500.12 ± 6592.54	0.33 ± 0.52	2707.18 ± 4421.64	0.10 ± 0.06	1165.38 ± 2706.66
**CON**	0.15 ± 0.08	2602.77 ± 4450.38	0.15 ± 0.18	273.92 ± 183.01	0.09 ± 0.05	146.59 ± 123.74

**Table 10:** Cortisol at 14 days and cortisol and alpha-amylase concentration at weaning of piglets from sows fed with electronic sow feeders (PF), feeding ball (FB), and traditional feeders (CON).

Group	14 Days Old	Weaning	
	Cortisol (μg/dL)	Cortisol (μg/dL)	Alpha-Amylase (U/L)
**PF**	4.57 ± 3.35 ^a^	0.15 ± 0.11	7803.24 ± 5632.77 ^g^
**FB**	2.75 ± 2.82 ^b^	0.14 ± 0.06	15,335.04 ± 9833.87 ^h^
**CON**	2.61 ± 3.26 ^b^	0.14 ± 0.08	6935. 06 ± 6275.03 ^g^

Different superscripts denote statistical differences (a ≠ b: *p* < 0.05; g ≠ h: *p* < 0.001).

**Table 11:** Mean values (±SD) and *p*-values for kg of feed per kg of weaned piglet and kg of feed per weaned piglet in sows fed with electronic sow feeders (PF), feeding ball (FB), and traditional feeders (CON).

Group	Kg of Feed per Kg of Weaned Piglet	Kg Feed per Weaned Piglet
**PF**	1.69 ± 0.21 ^a^	12.40 ± 1.05
**FB**	2.14 ± 0.50 ^b^	13.66 ± 1.96
***CON***	*2.05 ± 0.36*	*13.71 ± 1.59*

Different superscripts denote statistical differences (a ≠ b: *p* < 0.05).

## Data Availability

The original contributions presented in this study are included in the article. Further inquiries can be directed to the corresponding authors.
